# Supplementary material for: Continuous fish muscle cell line with capacity for myogenic and adipogenic-like phenotypes
Source: Sci Rep. 2023 Mar 29;13:5098. doi: 10.1038/s41598-023-31822-2 (PMC10060565; doi:10.1038/s41598-023-31822-2)
Supplement: Supplementary file 1 — Supplementary Information. [file 41598_2023_31822_MOESM1_ESM.pdf]

Supplementary Information

Supplementary Table S1: Media Formulations

Component	Concentration
**L-15 Growth Medium**	
Leibovitz's L-15 Medium (ThermoFisher Scientific #11415064)	
Fetal bovine serum (FBS, ThermoFisher Scientific #26140079)	20%
Recombinant human fibroblast growth factor (FGF-basic 154 a.a.; PeproTech #100-18B)	1 ng/mL
HEPES, pH 7.4 (Sigma Aldrich #H4034)	20 mM
Antibiotic-Antimycotic (ThermoFisher Scientific #1540062)	1%
Gentamicin solution (Sigma Aldrich #G1397)	10 µg/mL
**Adipogenic Medium**	
L-15 growth medium (described above)	
Insulin solution from bovine pancreas (Sigma Aldrich #I0516)	10 µg/mL
3-isobutyl-1-methylxanthine (IBMX, Sigma Aldrich #I5879)	0.5 mM
Dexamethasone (Sigma Aldrich #D4902)	0.25 µM
Lipid mixture (Sigma Aldrich #L5146)	10 µL/mL
**Reduced Serum Medium**	
Leibovitz's L-15 Medium (ThermoFisher Scientific #11415064)	
Fetal bovine serum (FBS, ThermoFisher Scientific #26140079)	2%
HEPES, pH 7.4 (Sigma Aldrich #H4034)	20 mM
Antibiotic-Antimycotic (ThermoFisher Scientific #1540062)	1%
Gentamicin solution (Sigma Aldrich #G1397)	10 µg/mL
+/- insulin-like growth factor 1 (IGF-1, Shenandoah Biotechnology #100-34)	100 ng/mL
**Reduced Serum + Additives**	
Reduced serum medium (described above)	
1-Oleoyl Lysophosphatidic Acid (LPA, Fisher Scientific #NC9401387)	10 µM
Optiferrin® recombinant human transferrin (InVitria #777TRF029)	135 nM
Insulin solution from bovine pancreas (Sigma Aldrich #I0516)	1.8 µM
+/- insulin-like growth factor 1 (IGF-1, Shenandoah Biotechnology #100-34)	100 ng/mL
**ERKi Medium**	
Reduced serum medium (described above)	
Calbiochem™ ERK Inhibitor (ERKi, Sigma Aldrich #328006)	2 µM

Supplementary Table S2: RT-qPCR Primers

Gene	NCBI Accession Number	Primer Direction	Primer Sequence (5' – 3')
HPRT	XM_042419962.1	Fwd	GTCTACGTTGACAGGCAAGAATGT
		Rev	GTCTGGTCGGTAGCCAACACT
PAX3B	XM_042415392.1	Fwd	AAGCGGGAAAACCCGGTGAG
		Rev	CCACATCCGAACCCTCGTCT
MYOD1	XM_042412988.1	Fwd	TTGGAGCACTACAGCGGGGA
		Rev	GCTGGTGTCGGTACTGATCCG
MYOG	XM_042406865.1	Fwd	GGAGCACCCTGATGAACCCC
		Rev	CGCTTGACGACGACACTCTGG
TNNT3A	XM_042412366.1	Fwd	TCAGCGCGGTAAGTTTGCAG
		Rev	CTCCTCTTCTACGGCCTCGACA
PPARG	XM_042402801.1	Fwd	CCATGCCTGTGAGGGCTGTA
		Rev	GGCCAAAACGAATGGCGTTG

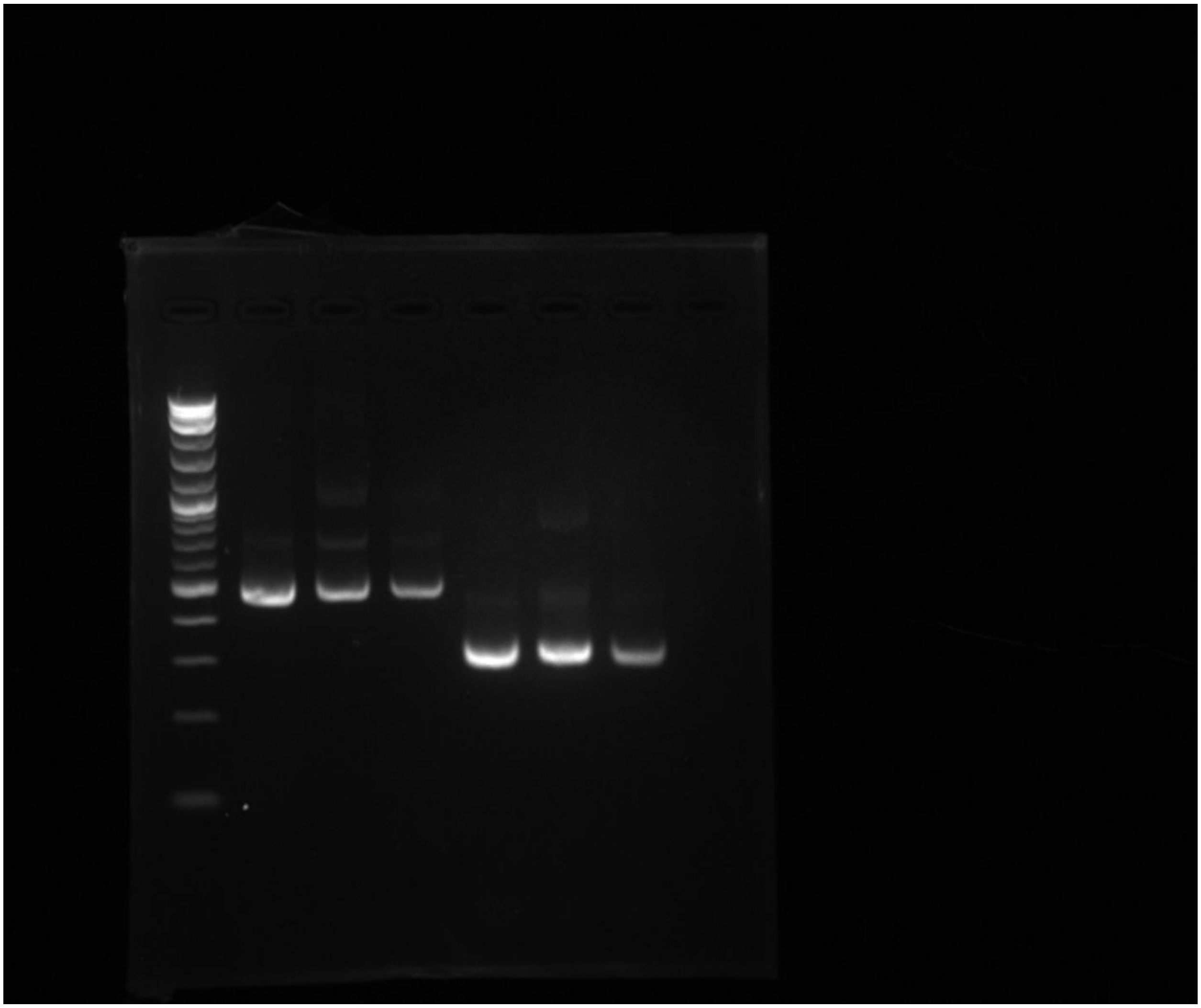

Supplementary Figure S1, related to Figure 1A: The uncropped gel image, from which Figure 1A was derived.

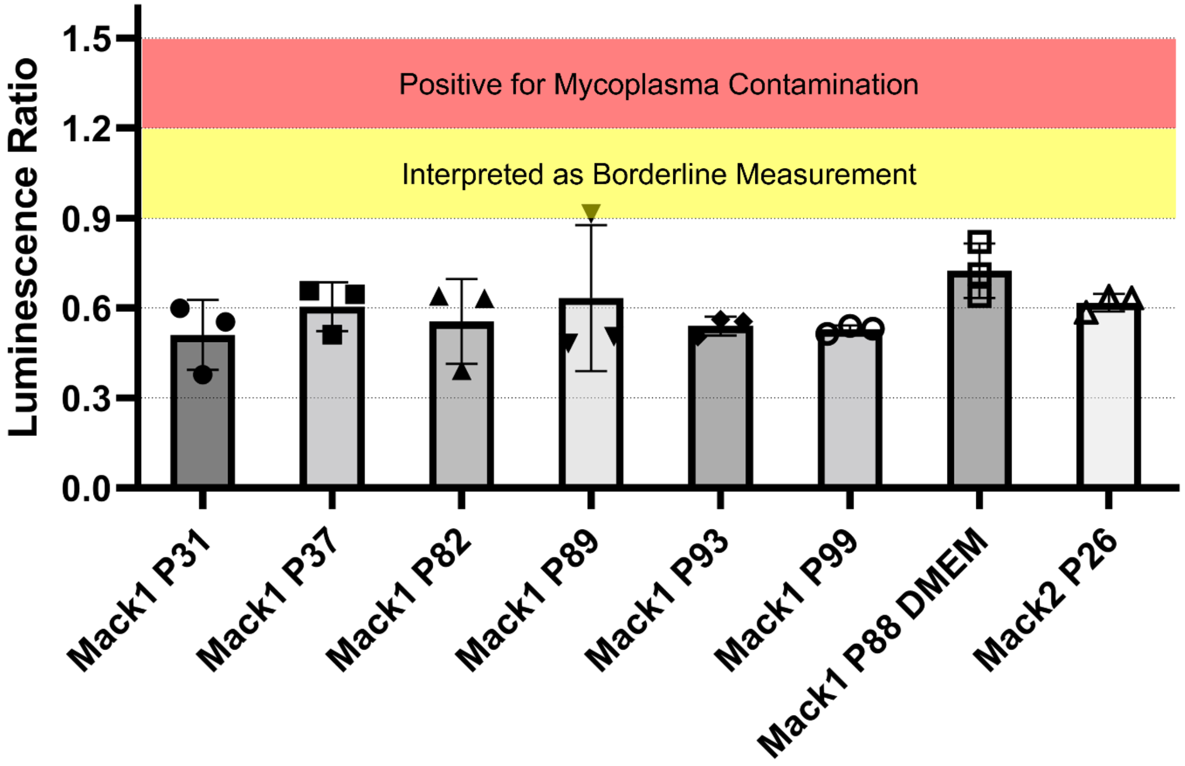

Supplementary Figure S2: Mycoplasma testing, as measured by MycoAlert® Mycoplasma Detection Kit. Luminescence ratio values between 0.9 and 1.2 indicate samples may be borderline for contamination, while values above 1.2 indicate contamination. Error bars indicate standard deviation, n =3.

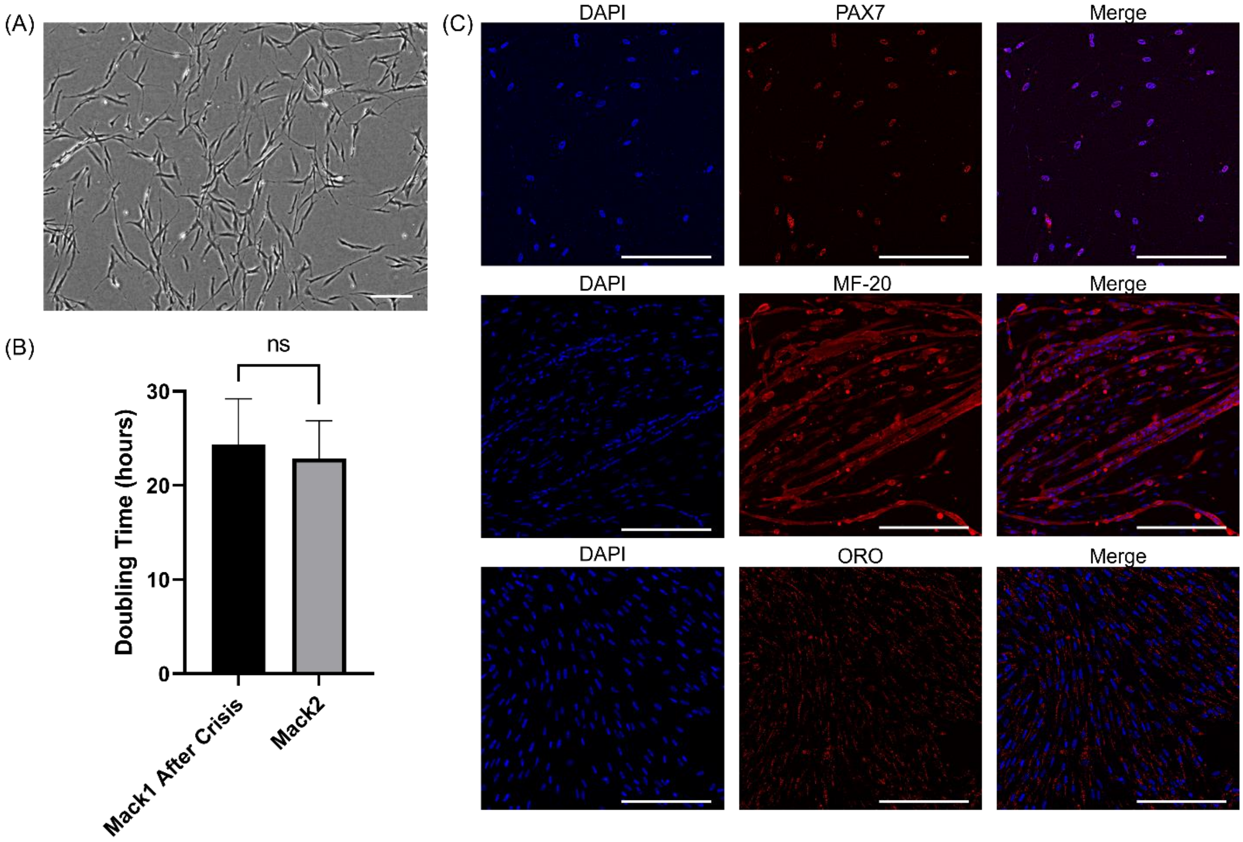

Supplementary Figure S3: Mack2 characterization. (A) Phase contrast imaging of Mack2 P31. Scale bar 200 µm. (B) Doubling time comparison between Mack1 after crisis (passage numbers 114-128) and Mack2 (passage numbers 39-53). Error bars indicate standard deviation, n = 15; statistical significance calculated by unpaired t-test, in which n.s. is not significant. (C) Immunofluorescent staining on Mack2 P27 for cell nuclei (DAPI, blue), PAX7 (red, top panel), MHC (MF-20, red, middle panel), and Oil Red O (red, bottom panel) during proliferation, muscle differentiation, and adipogenic-like differentiation, respectively. Scale bars 100 µm.

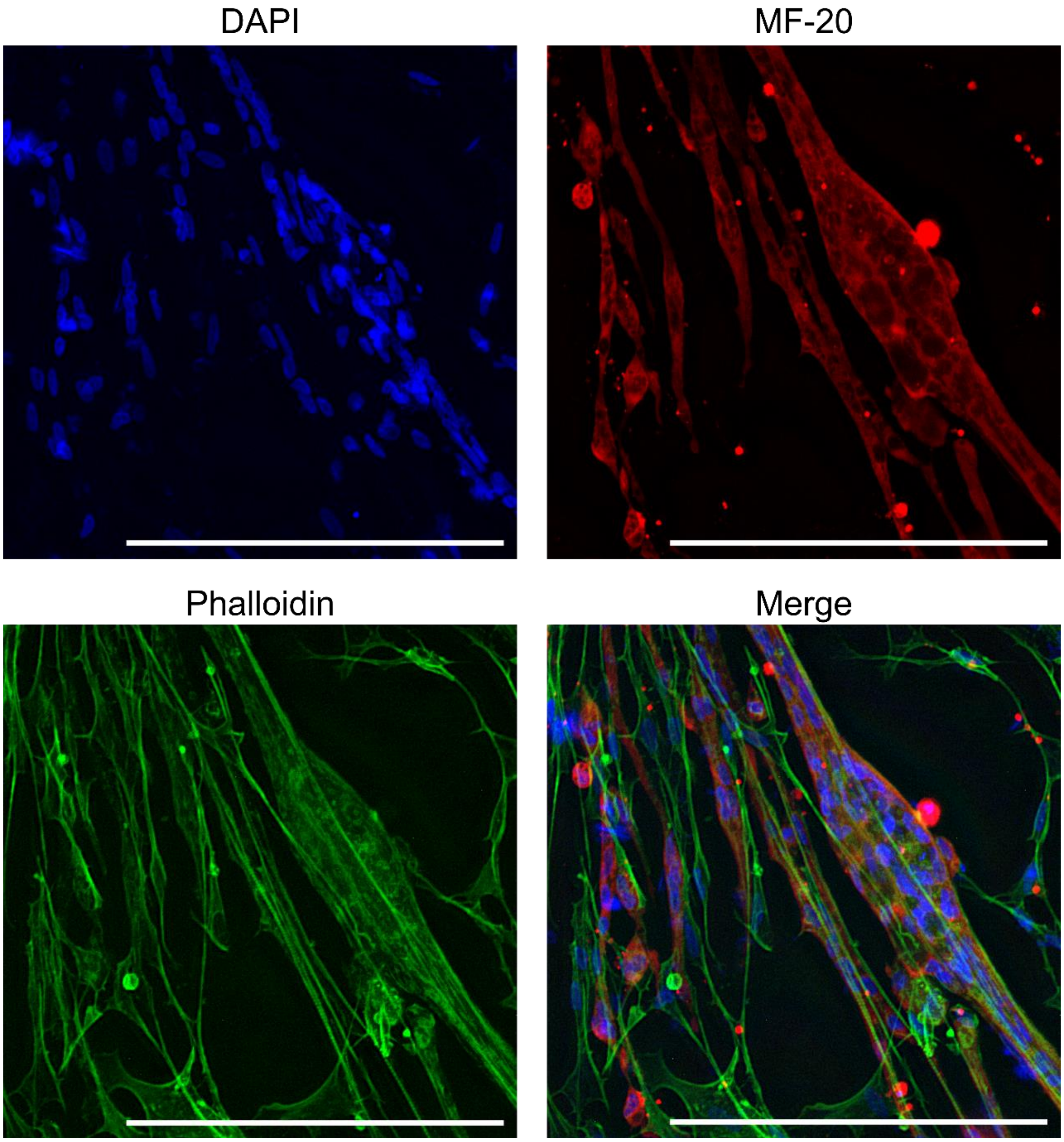

Supplementary Figure S4: Mack2 muscle differentiation. Immunofluorescent staining for cell nuclei (DAPI, blue, top left), MHC (MF-20, red, top right), F-actin (Phalloidin, green, bottom left), and merge of all three (bottom right). Scale bars 200 µm.

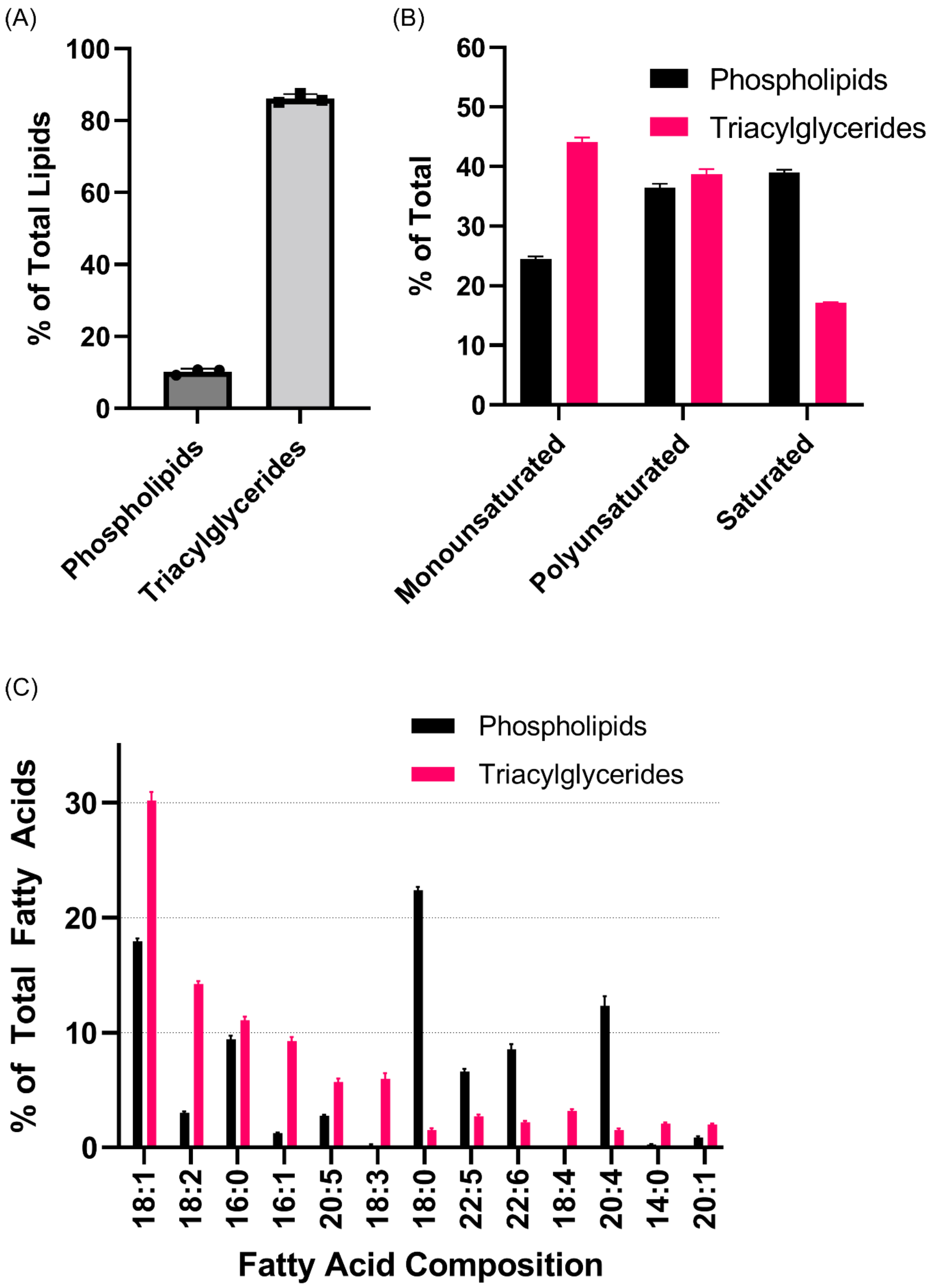

Supplementary Figure S5: Phospholipid (PL) and triaccylglyceride (TAG) lipidomics of adipogenic-like mackerel cells. (A) The percentage of PL and TAG, respective to the total lipids detected in lipidomics. (B) Percent of fatty acid saturation in the PL and TAG fractions. (C) The top 13 most prevalent fatty acids from the *in vitro* mackerel PL and TAG fractions are shown. Error bars indicate standard deviation, n = 3.
